# Goal-directed and stimulus-driven selection of internal representations

**DOI:** 10.1073/pnas.2013432117

**Published:** 2020-09-14

**Authors:** Freek van Ede, Alexander G. Board, Anna C. Nobre

**Affiliations:** ^a^Oxford Centre for Human Brain Activity, Wellcome Centre for Integrative Neuroimaging, Department of Psychiatry, University of Oxford, Oxford OX3 7JX, United Kingdom;; ^b^Institute for Brain and Behavior Amsterdam, Department of Experimental and Applied Psychology, Vrije University Amsterdam, 1081 HV Amsterdam, The Netherlands;; ^c^Department of Experimental Psychology, University of Oxford, Oxford OX1 3UD, United Kingdom

**Keywords:** visual working memory, attention, capture, oculomotor system, memory-guided behavior

## Abstract

Adaptive behavior relies on the selection of relevant sensory information from both the external environment and internal memory representations. In understanding external selection, a classic distinction is made between voluntary (goal-directed) and involuntary (stimulus-driven) guidance of attention. We have developed a task—the anti-retrocue task—to separate and examine voluntary and involuntary guidance of attention to internal representations in visual working memory. We show that both voluntary and involuntary factors influence memory performance but do so in distinct ways. Moreover, by tracking gaze biases linked to attentional focusing in memory, we provide direct evidence for an involuntary “retro-capture” effect whereby external stimuli involuntarily trigger the selection of feature-matching internal representations. We show that stimulus-driven and goal-directed influences compete for selection in memory, and that the balance of this competition—as reflected in oculomotor signatures of internal attention—predicts the quality of ensuing memory-guided behavior. Thus, goal-directed and stimulus-driven factors together determine the fate not only of perception, but also of internal representations in working memory.

## Significance

Everyday behavior relies on continuously selecting relevant information from the external environment as well as from internal representations in working memory. In describing the selection of external objects, a longstanding distinction is proposed between goal-directed (voluntary) and stimulus-driven (involuntary) sources of attentional selection. We voluntarily attend to things that are relevant to our goals, but our attention may also be captured involuntarily by salient events in the external world. Yet despite decades of research on how these two sources guide our perception (external selection), to date no study has examined whether and how these two sources jointly influence the selection of internal memory representations. With innovative experimental manipulations and behavioral markers of internal selection, we fill this important gap.

Everyday behavior as we know it relies on the continuous selection of relevant information from both the external environment and our internal representations within memory (1, 2). While many factors contribute to the allocation of attention (3–5), a prominent distinction in the literature on external attention is that between voluntary (goal-directed) and involuntary (stimulus-driven) sources of selection (6–10). We may voluntarily attend to a sensory stimulus because it is directly relevant to our goals, but our attention may also be captured involuntarily by stimuli in the external world. Attention can also be guided to select internal representations from working memory (11–13). Here we address whether goal-directed and stimulus-driven sources of selection similarly apply to and compete for selection of internal representations.

In working memory, a popular way to study attentional selection and prioritization is to present cues during the memory period that inform what information will become relevant for the upcoming task (12, 13). Such “retrocues” typically indicate the relevant memory item through one of its features (e.g., its visual location, color, or shape), while another memory feature is required for the task. Thus, retrocues enable the voluntary allocation of attention to goal-relevant memory content. At the same time, however, a match between a feature in the retrocue and in a memorandum may involuntarily lead to attentional allocation to the matching memory content, for example, via pattern completion (14). Such a potential “retro-capture” effect would be the reciprocal of when memory content triggers external attention to memory-matching sensory stimuli (15–20). Thus, typical retrocue findings may be due to both voluntary and involuntary attentional influences over the contents of memory.

To disentangle and examine voluntary and involuntary attentional influences on the contents of visual working memory, we developed the anti-retrocue task – inspired by the popular anti-saccade task (21), which has proven instrumental in disentangling voluntary and involuntary influences over the control of action (22). In our retrocue version of this task, we used pro- and anti-retrocues that were 100% predictive of the relevant memory item but differed in whether their visual feature matched the goal memory item (pro) or matched the other competing memory item (anti). Moreover, to isolate pure involuntary capture effects, we also included null blocks with retrocues whose visual features also matched either item but were otherwise uninformative, and we instructed participants to ignore these cues (unlike in previous studies using other types of uninformative cues) (23–25). Together, these conditions enabled us to disentangle and quantify the independent contributions of voluntary and involuntary attention over the contents of visual working memory.

Adding to this task innovation, we capitalized on our recent demonstration that attentional focusing in working memory is associated with directional biases in gaze behavior (26). Gaze biases provided us with a powerful tool to track attentional allocation to internal representations following both voluntary and involuntary influences and to address what happens when these two fundamental influences are present concurrently and compete for internal selection within working memory.

## Results

**Fig. 1.**
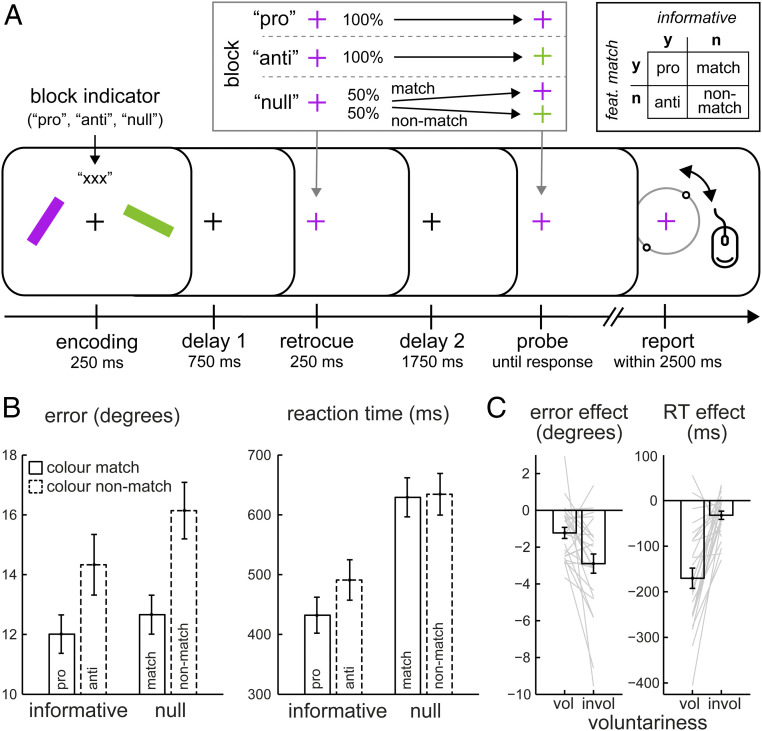
Voluntary and involuntary attention each influence memory but in distinct ways. (*A*) Task schematic. Participants remembered two visual tilted bars to reproduce the orientation of the probed memory item at the end of the delay. The probe was a lasting color change of the central fixation cross, while the retrocue color change was transient. Participants were always required to report the item indicated by the probe. The critical manipulation involved the attentional retrocue that was presented during the memory delay. We used four types of cues, yielding the two-by-two design (*Top Right*). Pro- and anti-retrocues were each 100% predictive of the relevant (to be probed) memory item but differed in whether their color also matched (pro) or nonmatched (anti) the to-be-probed item. Null-retrocues were not informative but also matched or nonmatched the subsequently probed memory item. (*B*) Behavioral performance as a function of whether the retrocue was informative (pro and anti) or not (match and nonmatch) and whether it matched the color of the probed memory item (pro and match) or not (anti and nonmatch). (*C*) Behavioral effects of voluntary and involuntary attentional influences on memory, with negative values corresponding to better performance (i.e., fewer errors and faster reactions) following informative cues (voluntary factor) or color-matching cues (involuntary factor). Error bars represent ±1 SEM, calculated across participants (*n* = 25). Gray lines depict individual participants.

Participants performed a visual working memory task (Fig. 1*A*) in which they memorized two colored tilted bars over a 3-s delay until a lasting color change of the central fixation cross (the probe) prompted them to reproduce the precise orientation of the corresponding memory item.

At 1 s into the memory delay, an attentional retrocue—a brief color change of the central fixation cross—indicated which of the bars would be probed after another 2 s. Retrocues carried different information in different blocks (Fig. 1*A*, *Inset*). In conventional “pro” blocks, cues predicted probes directly, such that a purple (green) retrocue indicated with 100% reliability that the corresponding purple (green) memory item would subsequently be probed for report. In contrast, in “anti” blocks, cues informed with 100% reliability that the other item would be probed for report—that is, a purple retrocue predicted that the green item would be probed, and vice versa. Finally, in “null” blocks, cues were not informative for which item would be probed, yielding 50% “match” and 50% “nonmatch” cues.

Across conditions, cues were therefore either informative (pro and anti) or not (match and nonmatch), and the cue’s feature could either match that of the probed memory item (pro and match) or not (anti and nonmatch). This unique two-by-two aspect of our design (Fig. 1*A*, *Top Right Inset*) enabled us to disentangle, and separately examine, voluntary influences of “goal-directed prioritization” and involuntary influences of “color capture” on the visual contents of memory.

### Voluntary and Involuntary Attention each Influence Working Memory, but in Distinct Ways.

Fig. 1*B* shows memory performance (reproduction errors and reaction times [RTs]) as a function of whether the retrocue was informative (voluntary factor), as well as whether its color matched the subsequently probed memory item (involuntary factor). For reproduction errors, the pattern of results revealed a particularly pronounced effect of the involuntary factor, whereby performance was better when the color of the probed memory item matched that of the cue (for both informative and null cues) (Fig. 1*B*, *Left*). This was confirmed by a highly robust main effect of color match (*F*_(1,24)_ = 31.055, *P* = 9.827e-6, η^2^ = 0.841). Although less apparent from the graph, cue informativeness also conferred a benefit (*F*_(1,24)_ = 16.488, *P* = 0.0005, η^2^ = 0.489), and color match and cue informativeness interacted (*F*_(1,24)_ = 5.097, *P* = 0.033, η^2^ = 0.175). The benefit of color match on performance was significant in both cases but was greater following uninformative retrocues (3.5° more accurate; *t*_(24)_ = −5.512, *P* = 1.145e-5, *d* = −1.102) than following informative retrocues (2.3° more accurate; *t*_(24)_ = −4.424, *P* = 1.797e-4, *d* = −0.885) (Fig. 1*B*, *Left*).

In contrast, RTs (Fig. 1*B*, *Right*) revealed a particularly pronounced effect of the voluntary factor. Reproduction reports were initiated sooner in trials with informative cues, both when cues matched the items in color and when they did not. This was confirmed by a highly robust RT benefit of cue informativeness (*F*_(1,24)_ = 58.084, *P* = 7.364e-8, η^2^ = 0.926), although we also found an RT benefit of color match (*F*_(1,24)_ = 12.686, *P* = 0.002, η^2^ = 0.306), as well as a significant interaction between the two factors (*F*_(1,24)_ = 7.459, *P* = 0.012, η^2^ = 0.237). The benefit of cue informativeness on RT was significant in both cases but was larger following color match retrocues (197 ms faster; *t*_(24)_ = −7.796, *P* = 4.975e-8, *d* = −1.559) than following color nonmatch retrocues (143 ms faster; *t*_(24)_ = −6.1, *P* = 2.663e-6, *d* = −1.22) (Fig. 1*B*, *Right*).

The pattern of results is best appreciated and quantified when directly comparing the effects of the voluntary and involuntary factors (Fig. 1*C*). The voluntary effect of goal-directed prioritization was obtained by comparing performance on trials with informative cues (pro and anti) and those with uninformative cues (match and nonmatch)s (i.e., the main effect of cue informativeness in our two-by-two operationalization), while the involuntary effect of color capture was obtained by comparing color matching cues (pro and match) vs. nonmatching cues (anti and nonmatch) (i.e., the main effect of color match in our two-by-two operationalization). For errors, the involuntary influence of color capture by the retrocue had a larger beneficial impact on performance than the voluntary influence of cue informativeness (2.9° vs. 1.2° more accurate; *t*_(24)_ = 2.773, *P* = 0.011, *d* = 0.555) (Fig. 1*C*, *Left*). In contrast, for RTs, the voluntary factor of cue informativeness had a greater beneficial impact (170 ms vs. 32 ms faster; *t*_(24)_ = −5.966, *P* = 0.011, *d* = −1.193) (Fig. 1*C*, *Right*).

### Voluntary and Involuntary Attention Bias Gaze toward Memorized Item Locations.

We recently reported a sensitive gaze-based signature of attentional focusing in visual working memory (26). During internal focusing, gaze becomes biased in the direction of the memorized location of a cued memory item, even when there is nothing to see at that location and even when item location is never asked about (as was also the case here). By measuring this “gaze bias” in the current task with pro-, anti-, and null-retrocues, we were in the unique position to address 1) whether the brain’s oculomotor system is similarly engaged for voluntary and involuntary attentional influences in working memory, 2) how each of these attentional influences unfolds in time, and 3) what happens when voluntary and involuntary attentional influences are present concurrently and compete for item selection in visual working memory.

**Fig. 2.**
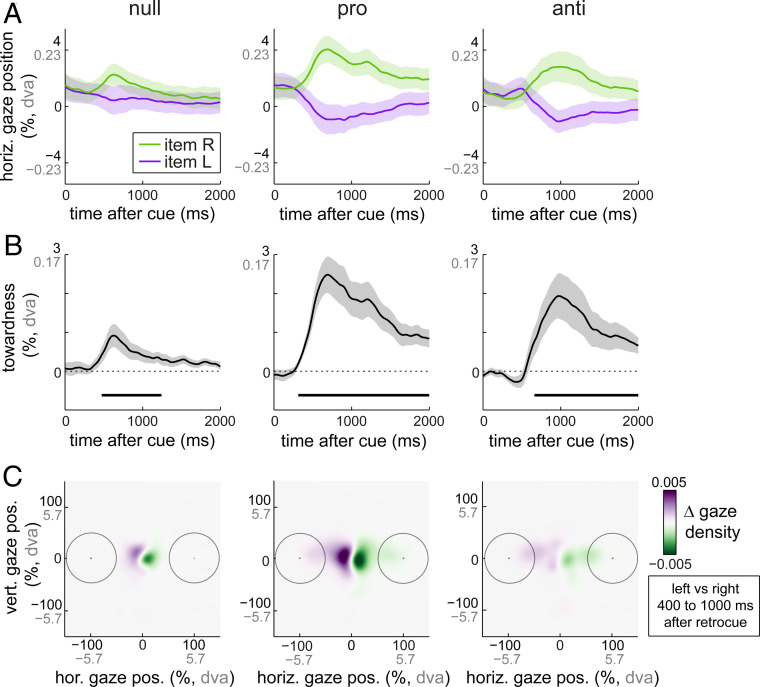
Voluntary and involuntary attention each bias gaze toward memorized item locations. (*A*) Horizontal gaze position biases following null-, pro-, and anti-retrocues to left and right memory items. Following null-retrocues, left and right are defined relative to the location of the color-matching item, whereas following pro- and anti-retrocues, left and right are defined relative to the location of the goal (to-be-probed) memory item. For completeness, we depict gaze values both in percentage towardness (with ±100% corresponding to the center of the memory items at encoding corresponding to a ±5.7° visual angle) as well as in degrees of visual angle (dva). (*B*) Gaze bias towardness time courses corresponding to the data in *A*. While the average gaze position bias is small in absolute magnitude (in line with a bias in fixational eye movements; ref. 26), it is also highly robust. Horizontal lines indicate significant clusters. (*C*) Heatmaps of the difference in gaze density following retrocues to left and right items. Circles indicate regions spanned by the memory items at encoding. Density maps were constructed by collating individual gaze samples in the 400- to 1,000-ms post-retrocue window, without averaging across time and trials. Shaded areas indicate ±1 SEM, calculated across participants (*n* = 25).

Fig. 2*A* shows the horizontal gaze position following cues to left and right memory items as a function of time after the retrocue, separately for null-, pro-, and anti-retrocues. Following null-retrocues, left and right are defined relative to the location (in memory) of the color-matching item, whereas following pro- and anti-retrocues, left and right are defined relative to the location of the goal (to be probed) memory item. Fig. 2*B* shows the associated “towardness” time courses, together with the significant clusters, corrected for multiple comparisons (27).

We first asked whether the pure bottom-up visual color feature of the cue is sufficient to bias gaze involuntarily to the matching memory item. In null-retrocue blocks (Fig. 2, *Left* column), we observed a clear gaze bias toward the memory item matching the color of the uninformative retrocue. If the central cue matched the color of the left memory item, gaze became biased to the left, whereas if the cue matched the color of the right memory item, gaze became biased to the right (Fig. 2*A*). This is also evident in the associated towardness time course (Fig. 2*B*; cluster *P* = 0.008).

A heat map of the difference in gaze density following left and right retrocues (constructed with individual gaze-position samples, collated over the 400- to 1,000-ms postcue interval) confirmed that this effect constituted a directional bias in gaze positions close to fixation, rather than full gaze shifts to the item’s location at encoding, in line with our previous report of this bias (26). Nonsubtracted heat maps of gaze density corroborated this interpretation, confirming an overall fixational focus in each condition (*SI Appendix*, Fig. S1).

**Fig. 3.**
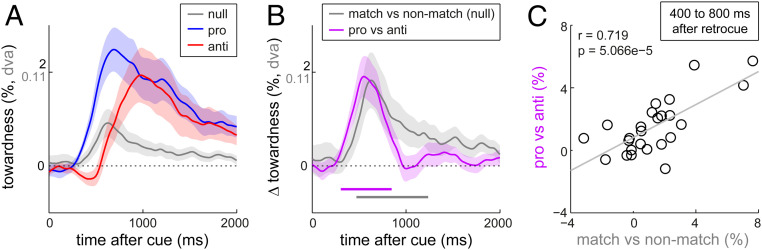
Voluntary and involuntary attention compete for item selection, as reflected in gaze. (*A*) Overlay of gaze bias towardness time courses following null-, pro-, and anti-retrocues (cf. Fig. 2*B*). (*B*) Overlay of the differences in gaze bias following null-retrocues to match vs. nonmatch items and the differences in gaze bias to the goal memory item following pro- vs. anti-retrocues. Note how the relevant match vs. nonmatch bias in null blocks is exactly double the size of the towardness measure in *A*, given that the relevant comparison here is with the difference in towardness toward a matching item vs. toward the opposite item. (*C*) Correlation across participants between the differences in gaze bias following null-retrocues to match vs. nonmatch items and the differences in gaze bias following pro- vs. anti-retrocues. Data are from the 400- to 800-ms postcue window in which both differences were pronounced in the group average (as in *B*). Shaded areas indicate ±1 SEM, calculated across participants (*n* = 25).

A similar but more pronounced gaze bias was observed following pro-retrocues (Fig. 2, *Middle* column; cluster *P* < 0.0001; pro vs. null cluster *P* < 0.0001; overlay in Fig. 3*A*). This shows that gaze bias is also sensitive to the voluntary deployment of attention to the relevant memory item.

What happens following anti-retrocues, when voluntary and involuntary influences are directed at opposite items? Following anti-retrocues (Fig. 2, *Right* column), we observed a predominant gaze bias to the goal (to be probed) memory item (cluster *P* = 3.999e-4), despite cues matching the color of the opposite memory item. This confirms that the voluntary influence is overall more potent in biasing gaze. At the same time, we saw an initial dip in the direction of the color-matching item only after anti-retrocues. Although this dip itself did not survive statistical evaluation (no clusters found), a careful examination of the respective time courses of these biases revealed an early competition between voluntary and involuntary attention, to which we turn next.

### Voluntary and Involuntary Internal Attention Compete for Internal Selection.

An overlay of the towardness time courses following null-, pro-, and anti-retrocues (Fig. 3*A*) reveals how gaze bias is delayed following anti-retrocues compared with pro-retrocues. This was corroborated by a jackknife analysis of the latencies at which gaze biases following pro- and anti-retrocues first reached 10% of their peak value toward the goal memory item (at 318 ± 21 ms and 567 ± 46 ms after the onset of pro- and anti-retrocues, respectively; jackknife *t*_(24)_ = 4.441, *P* = 0.0002). There are two potential explanations for this delay. First, it may simply take longer to interpret the anti-retrocues, stalling attentional deployment to the relevant memory item. Second, the process of voluntary deployment attention to the relevant memory item might co-occur (i.e., compete) with the involuntary color capture to the opposite item, yielding a net cancellation of the gaze bias early after the cue (when the capture effect is most pronounced). Indeed, as Fig. 3*A* shows, the overall timing of the involuntary capture effect in null blocks (reflecting the “pure” involuntary effect) largely overlaps with the time at which the gaze bias following pro- and null-retrocues start to differ (reflecting the “pure” voluntary influence). This suggests similar timing for internal selection through voluntary and involuntary influences, thereby making it viable for these two influences to be present concurrently and to counteract each other following anti-retrocues.

If the difference in gaze bias following pro- and anti-retrocues reflects the fact that voluntary and involuntary gaze biases add together in pro-blocks but compete in anti-blocks (the second scenario above), then the difference in gaze bias following pro- and anti-retrocues should match the difference in gaze bias toward matching vs. nonmatching items in null-blocks. We found two independent sources of evidence supporting this. First, the temporal profile (as well as the magnitude) of the match vs. nonmatch comparison was very similar to that of the pro vs. anti comparison (Fig. 3*B*; *r*_across_ _time_ = 0.79, *P* < 0.001). Second, participants with a greater color-match gaze bias in the null-retrocue blocks also showed a larger difference in gaze bias following pro- vs. anti-retrocues (Fig. 3*C*; *r*_(23)_ = 0.719; *P* = 5.066e-5). This provides good evidence that voluntary and involuntary attentional influences in visual working memory co-occur and directly compete for item selection (following anti-retrocues), and that this competition is reflected (and can be tracked) in oculomotor signatures linked to the internal focusing of attention.

Experiment 2 provided a third piece of evidence that the delayed gaze bias following color anti-retrocues reflects the competition between voluntary and involuntary factors rather than some peculiarity of using anti-retrocues per se. In Experiment 2 (presented in detail in *SI Appendix*), we used orientation (instead of color) retrocues and asked participants to reproduce memorized item color (instead of orientation). While we still observed robust voluntary cueing benefits and gaze biases in Experiment 2, involuntary capture was much less potent following the orientation-matching retrocues (*SI Appendix*, Fig. S2 *C* and *D*). Critically, now that we no longer observed a clear involuntary gaze bias following null-retrocues, we also no longer found a delay in the gaze bias to anti-retrocues (*SI Appendix*, Fig. S2*D*), corroborating that this delay (as observed in Experiment 1; Fig. 3*A*) is contingent on the concurrent presence of voluntary and involuntary influences.

### The Balance of Attentional Competition in Memory following Anti-Retrocues Predicts Performance.

**Fig. 4.**
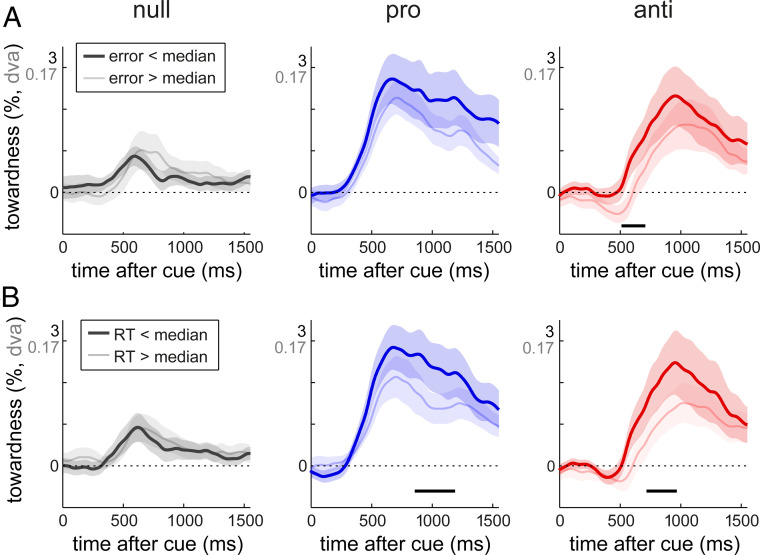
Competition between voluntary and involuntary influences predicts subsequent memory-guided performance. (*A*) Gaze bias towardness time courses sorted by behavioral accuracy (median split in reproduction errors) separately following null-, pro-, and anti-retrocues. (*B*) Gaze bias towardness time courses sorted by time from probe onset to response initiation. Horizontal lines indicate significant clusters, comparing gaze bias in trials with “good” (below the median) vs. “poor” (above the median) performance. Shaded areas indicate ±1 SEM, calculated across participants (*n* = 25).

To investigate whether the attentional operations following null-, pro-, and anti-retrocues—as tracked via gaze bias—influence performance, we split the data based on eventual memory performance and asked whether performance could be predicted by the pattern of gaze following the retrocue. Fig. 4*A* shows the data split for the quality of the reproduction report (below and above the median reproduction error), while Fig. 4*B* shows the data split by RT (below and above the median RT). Comparable results were obtained in Experiment 2 (*SI Appendix*, Fig. S3).

In null-retrocue blocks, we found no effect of the degree of involuntary capture on performance (Fig. 4, *Left* column). Thus, although we found clear capture effects on both behavior (Fig. 1*B*) and gaze (Figs. 2 and 3), we did not find that trial-to-trial variability in behavior was related to trial-to-trial variability in gaze bias to color-matching items. Possibly this is because this involuntary effect is relatively small and/or is associated with relatively little trial-wise variability. In contrast, in pro-retrocue blocks (Fig. 4, *Middle* column), we found that trials with better performance were associated with greater gaze bias, although this was significant only for RTs (cluster *P* = 0.032), in line with our previous study (26).

Critically, in anti-retrocue blocks (Fig. 4, *Right* column), gaze bias following the retrocue predicted both error (cluster *P* = 0.043) and RT (cluster *P* = 0.043). A smaller gaze bias to the goal (to be probed) memory item was associated with larger errors and slower responses to the subsequent probe. Following anti-retrocues, a smaller gaze bias to the voluntarily attended memory item may reflect a larger involuntary “pull” toward the other (color-matching) item. Indeed, in the trials with large (i.e., above the median) reproduction errors (Fig. 4*A*, *Right*, light-red line), gaze appears to be initially attracted to the “wrong” (color-matching) item, whereas this is not the case in trials with small (below the median) errors (Fig. 4*A*, *Right*, dark-red line). Although this initial bias to the wrong item did not survive correction (cluster *P* = 0.078), it did contribute to the significant difference between trials with small and large errors.

Thus, although the gaze bias following color-matching retrocues does not appear to predict performance by itself (in null-retrocue blocks), when voluntary and involuntary influences compete (in anti-blocks), performance appears to be related to the balance of this competition in early stages after the retrocue.

Finally, a key feature of the gaze bias following anti-retrocues was that the bias (toward the goal memory item) was delayed compared with that following pro-retrocues (Fig. 3*C*), suggesting that it takes time to overcome competition from the involuntary capture. A similar delay was found when comparing trials with small errors and those with large errors within the anti-retrocue condition (reaching 10% of the peak value at 523 ± 39 ms vs. 624 ± 47 ms, respectively; jackknife *t*_(24)_ = 2.459, *P* = 0.022). Thus, attentional allocation to the goal memory content is delayed in the presence of involuntary influences (Fig. 3*A*), and the degree of this capture-induced delay predicts the quality of ensuing memory-guided behavior (Fig. 4*A*, *Right*).

## Discussion

Our data reveal that both voluntary and involuntary factors contribute to attentional selection within the internal space of visual working memory, but with dissociable effects on performance. Voluntary (goal-directed) attention predominantly affects memory accessibility or “readiness” to act on the selected representation (as reflected in faster response-onset times after the probe following informative retrocues vs. uninformative retrocues), while involuntary (stimulus-driven) attention predominantly affects memory quality (as reflected in smaller degrees of error following color-matching vs. nonmatching retrocues)—a pattern distinct from the behavioral dissociations between voluntary and involuntary attention in external attention (28). We speculate that the involuntary retro-capture effects in memory are driven primarily by a process of automatically “refreshing” (29) the feature-matching memory item (14, 23, 24), benefiting memory quality without affecting memory accessibility or readiness, while voluntary effects are driven primarily by also placing the instructed memory item in an state of direct access, ready for upcoming task demands (11, 20, 30).

Numerous previous studies on the interaction between visual working memory and attentional capture have revealed how the contents of visual working memory can automatically (involuntarily) orient attention to matching stimuli in the external world (15–20). Here we provide evidence for a reverse capture (retro-capture) effect, whereby stimuli in the external world can also automatically trigger attention to matching internal sensory representations in working memory. Moreover, by capitalizing on subtle directional biases in human gaze behavior (31–33), we were able to directly track the associated attentional allocation to the feature-matching memory item. These automatic capture findings are in line with previous studies on visual working memory that used uninformative cues (23–25), with the key difference that in our task, participants were instructed to ignore the feature-matching cues (null-retrocues) or even to attend away from the feature-matching item (anti-retrocues), rather than to reflect on the cue (25) or on the associated memory item (23, 24).

Following anti-retrocues, we found evidence for a delayed attentional allocation (gaze bias) to the goal memory item, consistent with previous studies on the use of “negative cues” for upcoming perceptual tasks (34, 35). Our data not only show how participants can also use negative cues to select the appropriate memory content (see also refs. 36–39), but also suggest how the delayed attentional allocation to the appropriate item reflects the time required for involuntary and voluntary factors to resolve their competition at early stages after the retrocue and with consequences on ensuing memory-guided behavior. Specifically, we provide several complementary pieces of evidence indicating that the concurrent competition between voluntary and involuntary sources of selection contributed to the observed delay. First, the difference in gaze bias following pro- and anti-retrocues could be largely accounted for by the pure capture effect in the null blocks, in terms of its profile, timing, and magnitude as well as its variability across participants. Second, we no longer observed this delay in Experiment 2 with anti-retrocues that introduced little capture (*SI Appendix*). Finally, anti-retrocue trials with poorer performance (putatively trials that had more competition) were also associated with a delayed gaze bias. At the same time, we cannot rule out the possibility of additional factors contributing to this delay, such as extra time to process the cue and/or increased cognitive load associated with anti-retrocues.

Building on previous studies that used symbolic directed-forgetting cues to direct attention away from items in working memory (e.g., refs. 36–39), our anti-retrocues were designed such that they always matched the visual feature of one item while directing goal-based attention to the other item. By manipulating the feature match and instruction of the retrocue, it was possible to reveal the automatic capture of internal representations by (visually matching) external stimuli and also to track internal selection when voluntary and involuntary sources were in direct competition. In contrast, previous directed-forgetting studies tended to rely on other types of cues, such as arrow symbols (39) or word cues (37). Whether such cues also initially draw attention to the instructed set of items to be ignored—possibly accounting for the reduced benefit and neural modulation associated with directed-forgetting (as opposed to directed-remembering) cues (37)—remains an interesting possibility.

In our experiment, the voluntary factor (goal item) and the involuntary factor (feature-matching item) map neatly onto the distinction between goal-directed and stimulus-driven attention. There are also other, non–stimulus-driven influences over working memory that could be categorized as “involuntary.” For example, recent studies have shown how manual and saccadic actions, planned and/or executed during memory delays, can facilitate memory performance of items at action-congruent locations (40, 41). This occurs even when these items are less likely to be probed (41), reflecting automatic “action-driven” selection. Serial order effects (e.g., recency effects) provide another example (42, 43). Like our involuntary stimulus-driven effects, these involuntary influences provide additional influences over working memory that can occur alongside voluntarily attending the goal memory item (43).

Our data reveal that despite their dissociable effects on behavior, both voluntary and involuntary factors are associated with directional biases in human gaze behavior within memorized visual space (despite there being nothing to look at and location never being probed). This extends our previous demonstration of the existence of this “gaze bias” (26), which had relied only on conventional pro-retrocues, leaving the relative contributions of voluntary and involuntary factors unaddressed. Here we show that both factors bias gaze, and that the competition between these factors (as reflected in these oculomotor measures of covert attention) predicts subsequent memory performance. These observations reinforce and extend the utility of this time-resolved gaze bias as a tool for tracking internal attention, showing that this bias can be used to track multiple types of internal attention and that it captures processes that are behaviorally relevant. By implicating the brain’s oculomotor system in the allocation of both voluntary and involuntary attention within memory, these data also extend previous work linking the oculomotor system to attention (44, 45) and visual working memory (26, 46).

We have proposed that our involuntary retro-capture effects may be driven primarily by a process of refreshing memory contents without necessarily changing their accessibility for guiding behavior, while our voluntary effects may be driven primarily by placing the instructed memory item in a state of direct access, ready to guide upcoming behavior. Key evidence for this speculation is provided by the prominent facilitation of RTs following informative cues. In future work, it would be of interest to assess this more directly using electrophysiological or neuroimaging measurements. For example, by linking the memory items to specific manual actions (47), one could use such measurements to assess whether only informative cues would trigger the selection and subsequent preparation of the appropriate action in anticipation of the probe, while uninformative cues may affect primarily the representational fidelity (decodability) of the matching memory items.

Finally, by bringing the logic of the popular anti-saccade task (21, 22) to the domain of working memory, the present anti-retrocue task provides a powerful and elegant approach for disentangling voluntary and involuntary influences on memory. By placing voluntary and involuntary influences in competition, this also enables us to study a common (and thus relevant) situation often faced by our cognitive system outside the laboratory, where these distinct influences are abundant and often compete. As our data reveal, such competitive dynamics between goal-directed and stimulus-driven factors determine the fate not only of perception (15, 18), but also of internal representations in memory.

## Methods

The experimental procedures were reviewed and approved by the Central University Research Ethics Committee of the University of Oxford.

### Participants.

Sample sizes for the main Experiment 1 and Experiment 2 (*SI Appendix*) were set to 25 before data collection, based on previous studies from our laboratory that used similar tasks with similar outcome measures. For Experiment 1, all 25 participants (age range 22 to 31 y, 12 females, all right-handed) were retained for analysis. For Experiment 2, we had to recruit a total of 29 participants (age range 20 to 33 y, 14 females, all right-handed), because 4 participants had to be replaced to yield the intended sample of 25 (age range 20 to 33 y, 12 females). One participant was replaced because the eye-tracking data were of too-poor quality, and three participants were replaced because a mixture-modeling analysis showed that these participants reported the correct memory item in <50% of the anti-cue trials (compared with a mean of 91.996 ± 0.021% in the remaining participants and 88.583 ± 0.023% in Experiment 1). All participants were healthy human volunteers who provided written informed consent before participation. Participants were compensated at £10/h.

### Task and Procedure, Main Experiment 1.

The basic memory task was similar to that in several previous studies from our laboratory (e.g., ref. 26) and was programmed in Presentation. Participants memorized two colored and oriented bars to reproduce the orientation of one of the bars after a memory delay (Fig. 1*A*).

Participants sat ∼95 cm in front of a monitor on which the task was displayed (22-inch; resolution, 1,680 × 1,050 pixels; refresh rate, 100 Hz). Memory items involved visual bars that were 5.7° visual angle in width and 0.8° in height and were centered at a 5.7° visual angle to the left and right of fixation. In Experiment 1, each trial contained one green bar (RGB: 133, 194, 18) and one purple bar (RGB: 197, 21, 234) at randomly drawn orientations (180 possible orientations).

Visual bars were presented for 250 ms, followed by a blank in which only the fixation cross (spanning a surface area of 0.4° in total width and height) remained on the screen. At 1 s after encoding onset, a retrocue was presented for 250 ms that informed which item would subsequently be probed for report. The retrocue involved a brief change in the color of the central fixation cross. At 2 s after retrocue onset, the memory probe was presented that prompted participants to report the probe-matching memory item. Like the retrocue, the probe consisted of a change in color of the central fixation cross; however, unlike the retrocue, the change in color of the fixation cross at the probe stage was not brief, but lasted until the report was completed. Participants were instructed to reproduce the precise orientation of the memory item whose color matched that of the central probe. Participants were never explicitly asked about item location.

After probe onset, participants had unlimited time to initiate their report but were required to complete their orientation reproduction report (“dial-up”) within 2,500 ms after response initiation. The response dial appeared at response initiation at a random angle and always appeared centrally around the fixation cross, with the same diameter as the bars. Two small handles on the dial indicated the current reporting position on the dial. Dial-up was performed with the computer mouse, operated with the dominant (right) hand. Participants initiated the dial-up by moving the mouse and terminated the dial-up by clicking the mouse button with their index finger. At response termination, participants received feedback in the form of a number that indicated the quality of the report, scaled between 1 and 100 (1 reflecting the maximum error of 90° and 100 reflecting a perfect report). Feedback was presented for 200 ms and was followed by a randomly assigned intertrial interval of 500 to 800 ms.

The key manipulation was that retrocues carried different information in different blocks, referred to as “pro,” “anti,” and “null” blocks. In pro-blocks, retrocues were 100% informative that the same (color-matching) item would be probed later. A green retrocue predicted a green probe, while a purple retrocue predicted a purple probe. In contrast, in anti-blocks, cues informed with 100% reliability that the other item would be probed for report. A green retrocue now informed that the purple item would be probed, and vice versa. Finally, in null-blocks, cues were not informative for which item would be probed, yielding 50% “match” and 50% “nonmatch” cues. In match retrocue trials, the cue color happened to match that of the subsequently probed memory item, whereas in nonmatch retrocue trials, the cue color happened to match the other (nonprobed) memory item.

In addition to pro-, anti-, and null-retrocues, we included “neutral” retrocues in a minority of trials (25%). Neutral cues involved a color change to gray. These trials were not included in the main analyses presented here for two reasons, besides their relatively low trial numbers. First, for analysis of the behavioral data, we focused on the unique two-by-two nature of our design, by which cues could be either informative or not (pro and anti vs. match and nonmatch), while the cue’s visual feature (color) could either match the probed memory item or not (pro and match vs. anti and nonmatch). Neutral cues have no place in this two-by-two operationalization. Second, for our analysis of the eye-tracking data, we focused on the directional measure of “gaze bias” (26). This measure is defined relative to pro-, anti-, match-, and nonmatch-retrocues but not relative to neutral cues with no relation to left/right memory items. Nevertheless, for transparency and completeness, we present the behavioral data from these neutral retrocues alongside the data from our four main conditions in *SI Appendix*, Fig. S4.

Each experiment contained 15 pro-, 15 anti-, and 15 null-blocks with 16 trials in each, yielding a total of 720 trials and lasting ∼90 min. Before each block, the screen informed the ensuing block type by displaying the word “pro,” “anti,” or “null.” Participants started each block themselves by pressing ENTER on the keyboard in front of them. Moreover, to avoid potential confusion, we also displayed the block type at the top of the screen, where it remained visible throughout the block (indicated only on the first screen in Fig. 1*A*). Participants were explicitly informed what the block types meant, and how the cues should be used (in pro- and anti-blocks) or ignored (in null-blocks) in each block type.

After every three blocks (containing one block from each block type, in random order), we inserted a custom gaze calibration module in which participants were asked to look at a small white calibration square that was repositioned every 1 to 1.5 s among seven 7 positions (left-top, left-middle, left-bottom, right-top, right-middle, and right-bottom, as well as the center of the screen). Positions were visited in randomized order, with three visits per position per module. Calibration positions were set at a distance of 5.7° visual angle from the center of the screen, such that the left-middle and right-middle calibration positions corresponded to the centers of the memory items in the primary task.

Before the start of the experiments, participants practiced on one block from each block type. Practice always started with a pro-block, followed by an anti-block and then a null-block.

### Task and Procedure, Experiment 2.

Experiment 2 (*SI Appendix*) was identical to Experiment 1 with the key difference that participants were asked to reproduce precise item color (instead of orientation) while items were retrocued and probed through their orientation (instead of color). To this end, trials in Experiment 2 always contained one vertical bar and one horizontal bar at randomly drawn colors (180 possible values from the CIELAB color space), and the retrocues and probes consisted of a vertical or horizontal line (*SI Appendix*, Fig. S2). Participants performed a similar report as in Experiment 1, dialing-up color on a circular symmetrical color wheel. To disambiguate orientation retrocues from the fixation marker, we substituted the fixation cross from Experiment 1 with a fixation square (0.06° width and height) in Experiment 2.

### Randomization.

Recruitment for Experiments 1 and 2 was performed independently, although participants could participate in both. Each experiment consisted of a within-subjects design in which both block type and the location of the cued memory item were varied. Block type was randomized through 15 superblocks each containing one pro-block, one anti-block, and one null-block in random order. The location of the cued memory item was randomized within blocks, while ensuring an equal number of left- and right-item trials in each block. Item color and orientation were varied independently and were each independent from item location.

### Eye Tracking.

The eye tracker (EyeLink 1000; SR Research) stood ∼15 cm in front of the monitor on a table. Horizontal and vertical gaze position were continuously acquired from both eyes, at a sampling rate of 1,000 Hz. The eye tracker was calibrated and validated before the experiment using built-in protocols from the EyeLink software.

After acquisition, eye tracker data were converted from their original .edf format to the .asc format and read into MATLAB using FieldTrip (48). We used custom code to detect blinks and interpolated the signal from ±100 ms around blinks using a spline interpolation. We averaged data from the left and right eyes for both the horizontal and the vertical gaze position channels.

We used the data from the custom calibration modules to normalize the gaze position data in the task. We did this separately for each participant. For normalization, we calculated the median gaze position values at each of our seven calibration positions in the window of 500 to 1,000 ms after calibration point onset. We scaled our data such that these values corresponded to ±100% (corresponding to a ±5.7° visual angle). Because our gaze calibration positions were chosen to match the eccentricity of the center of the memory items in the task, ±100% in the horizontal gaze signal corresponded to the center of the memory items, after normalization.

Our gaze analyses focused on gaze position as a function of time. We compared conditions in which the cued memory item had occupied the left or the right item position during visual encoding. In accordance with our previous study (26), we also constructed a measure of “towardness” that expressed gaze bias toward the memorized item location in a single value per time point, thereby increasing sensitivity and interpretability and making it easier to visualize the comparison of this bias between the conditions of the experiment. Towardness was calculated by comparing the horizontal gaze position following cues associated with right minus left memory items, divided by 2. In null-blocks, left and right were defined relative to which memory item matched the visual feature (color in Experiment 1, orientation in Experiment 2) of the retrocue, whereas in pro- and anti-blocks, left and right were defined relative to which memory item was instructed to be the relevant (i.e., to be probed) item. Trial-average gaze position time courses were smoothed by a Gaussian kernel with an SD of 25 ms.

Our previous work showed that the gaze bias phenomenon is constituted by a bias in gaze around fixation (in line with refs. 31–33). Using this knowledge to our advantage, we deliberately concentrated the current gaze time course analyses on trials in which gaze position remained within a reasonable range of fixation. To remove the potential contribution of larger gaze shifts (as well as noise resulting from the interpolation stage, or measurement noise), we only included trials in which normalized gaze position values remained within ±50% from fixation (with 100% denoting the original item locations at a ±5.7° visual angle) throughout the course of the trial. This was the case in the vast majority of trials (Experiment 1, 92.8 ± 2.3%; Experiment 2, 93.0 ± 2.0%).

For visualization purposes, and to corroborate the fixational nature of the gaze bias, we also constructed heat maps of gaze density, without removing trials with gaze values beyond ±50%. We constructed two-dimensional (2D) histograms of gaze position by collating gaze position values across time and trials (without averaging) using the data from the 400- to 1,000-ms window after the retrocue. We chose this window because we had used it for the same purpose previously (26), and because the gaze bias was pronounced in this window in all relevant conditions. Counts were obtained at a 1% × 1% spacing, in normalized space. We divided 2D gaze position counts by the total number of gaze position samples to yield a density. Density maps were smoothed by a 2D Gaussian kernel with an SD of 10%. To depict the heat map associated with the directional gaze biases of interest, we subtracted maps between conditions in which cues were associated with left items vs. right items.

### Statistical Evaluation.

Our primary statistical analyses involved condition comparisons of behavioral and gaze position data within each experiment, where we could rely on powerful within-subject comparisons. For analysis of the behavioral data, we considered two measures: reproduction errors and response times. Reproduction errors were defined as the absolute difference between the probed memory item’s orientation (Experiment 1) or color (Experiment 2) and the reported orientation or color, as each defined in the semicircular 180° space. Response time was defined as the time from probe onset to response initiation. Trials with response times with a *z*-score larger or smaller than ±4 (Experiment 1, 0.919 ± 0.143%; Experiment 2, 0.783 ± 0.084%) were removed from the analysis, following a maximum of three iterations.

Across conditions, cues were either informative (pro and anti) or not (match and nonmatch), and the cue’s feature could either match that of the probed memory item (pro and match) or not (anti and nonmatch). This two-by-two aspect of our design enabled us to independently quantify voluntary influences of “goal-directed prioritization” and involuntary influences of “color capture” (Experiment 1) or “orientation capture” (Experiment 2) on memory performance. To this end, we used repeated-measures ANOVA with factors cue informativeness and cue color/shape match. In addition, we directly compared the voluntary effect of cue informativeness with the involuntary effect of cue color/orientation match using paired-samples *t* tests and compared voluntary and involuntary effects between experiments using independent-samples *t* tests.

For the statistical evaluation of our gaze position time courses, we used cluster-based permutation analyses (27), as in our previous work (26). This approach allowed us to evaluate effects in extended data (here extended in time) while elegantly circumventing the multiple-comparisons problem that otherwise would be faced. We used the default cluster settings in FieldTrip (48) and ran 10,000 permutations per evaluation. We focused our inferential statistical evaluations on the towardness time courses of gaze position. Complementary analysis of gaze density served only to characterize and confirm the nature of the gaze position bias and was not subjected to further statistical evaluation.

Pearson correlations were used to relate the difference in gaze bias following pro- vs. anti-retrocues with the difference in gaze bias following match vs. nonmatch cues in the null blocks. We did this once across time points (from 0 to 2,000 ms postcue) and once across participants. For the correlation across participants, we first averaged the data over the window from 400 to 800 ms, in which the gaze bias differences between conditions were most pronounced (in the group average).

A jackknife approach (49) was used to quantify latency differences in the onset of the of gaze towardness time courses following pro- and anti-retrocues. To this end, we estimated the time at which each towardness time course first reached 10% of its peak value. We obtained a jackknife estimate of the reliability of the onset latencies (as well as the differences in onset latency between cueing conditions) by iteratively removing participants from the pool and comparing the resulting latency difference with the observed difference in the full sample. The resulting jackknife estimate of the SE allowed us to evaluate the latency difference against the null hypothesis of no difference using the standard t-distribution.

A median-split analysis was used to investigate the relationship between gaze bias after the retrocue and subsequent performance after the probe. We applied a median split separately on errors and response times (and separately for each condition in the experiment) and calculated gaze towardness time courses for “good” (below the median) and “poor” (above the median) performance trials. We used cluster-based permutation analysis to compare gaze bias between trials with good and poor performance.

All reported measures of spread entail ±1 SEM, calculated across participants. Inferences were two-sided at an α level of 0.05.

## Supplementary Material

Supplementary File

## Data Availability

Data from both experiments have been made publicly available at https://doi.org/10.5281/zenodo.3996588.
