# Supplementary material for: Goal-directed and stimulus-driven selection of internal representations
Source: Proc Natl Acad Sci U S A. 2020 Sep 14;117(39):24590–8. doi: 10.1073/pnas.2013432117 (PMC7533705; doi:10.1073/pnas.2013432117)
Supplement: Supplementary File [file pnas.2013432117.sapp.pdf]

SUPPLEMENTARY INFORMATION (SI APPENDIX)

Supplementary information associated with the manuscript:

title: Goal-directed and stimulus-driven selection of internal representations
authors: Freek van Ede, Alexander Board, Anna C Nobre
journal: Proceedings of the National Academy of Sciences USA (PNAS)
year: 2020
contact: freek.van.ede@vu.nl

Supplementary Figure 1

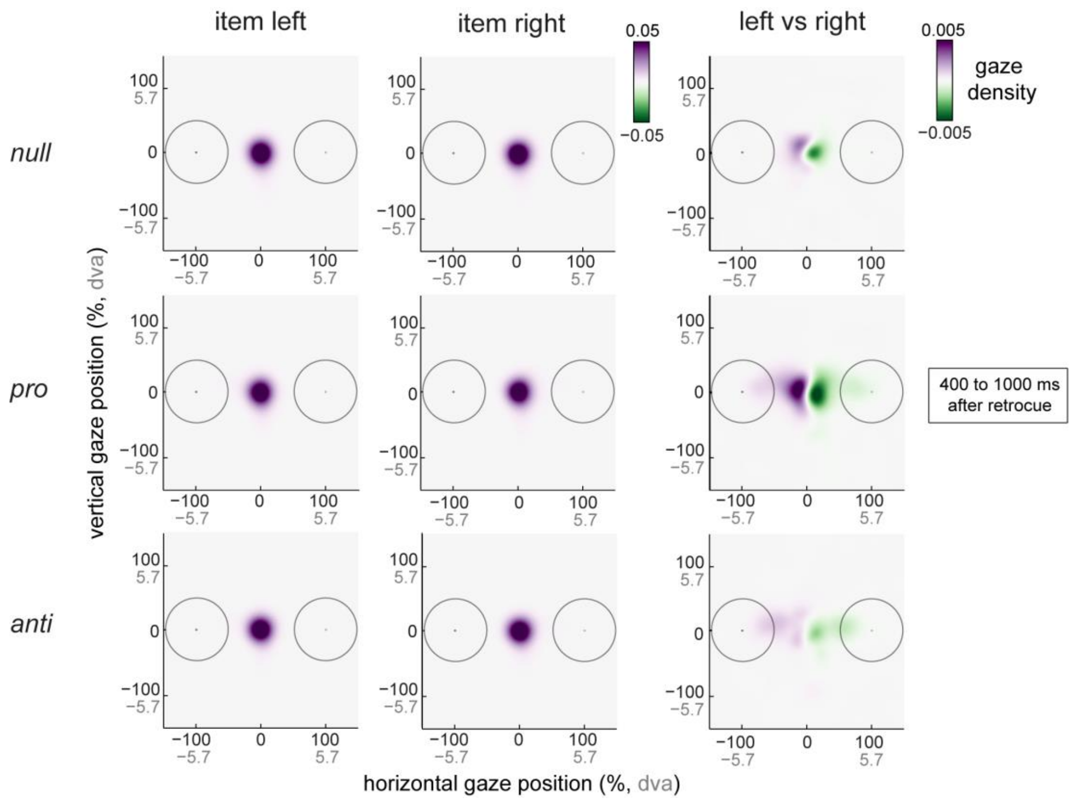

Figure S1. Heat maps of gaze density before left-right subtraction in Experiment 1. Gaze density maps before and after subtraction. Subtracted data in the right column correspond to the data in **Figure 2c**.

www.pnas.org/cgi/doi/10.1073/pnas.2013432117

Supplementary Experiment 2 (with Figures S2-S3)

Experiment 2 followed the exact same procedure as Experiment 1, except that we used retrocues and probes of a specific orientation (instead of colour), and asked participants to reproduce memorised item colour (instead of orientation) (**Fig. S2a**; see also Methods for details).

For error (**Fig. S2b**, left), we again found a main effect of orientation-matching ($F_{(1,24)}$ = 13.589, p = 0.001, η^2 = 0.605), and a main effect of retrocue informativeness ($F_{(1,24)}$ = 31.085, p = 9.762e-6, η^2 = 0.735), but no significant interaction ($F_{(1,24)}$ = 0.681, p = 0.417, η^2 = 0.028). For reaction times (**Fig. S2b**, right), we also found a main effect of orientation-matching ($F_{(1,24)}$ = 10.225, p = 0.004, η^2 = 0.396) and of cue informativeness ($F_{(1,24)}$ = 37.165, p = 2.690e-6, η^2 = 0.934), as well as an interaction between them ($F_{(1,24)}$ = 4.827, p = 0.038, η^2 = 0.168), whereby the orientation-match effect on RT was more pronounced in trials with informative cues (**Fig. S2b**).

Involuntary retro-capture by orientation-matching retrocues in Experiment 2 was less pronounced than involuntary retro-capture by colour-matching cues in Experiment 1. When directly comparing experiments: the pronounced involuntary colour retro-capture effect on error in Experiment 1 was significantly larger than the orientation retro-capture effect in Experiment 2 (2.9 vs. 1.4 degrees more accurate; **Fig. S2c**, left; $t_{(48)}$ = -2.279, p = 0.027, d = -0.6447). In contrast, the pronounced facilitatory effect of the voluntary factor on reaction times that we identified in Experiment 1 was highly comparable between experiments (170.2 vs. 192.6 ms faster following informative vs. null retrocues; **Fig. S2c**, right; p = 0.566). In accordance, in Experiment 2, voluntary and involuntary factors no longer differed significantly in their influence on reproduction errors ($t_{(24)}$ = -1.268, p = 0.217, d = -0.254), while reaction time benefits were still much more pronounced for the voluntary factor ($t_{(24)}$ = -5.332, p = 1.780e-5, d = -1.066).

In line with this reduced retro-capture effect by orientation retrocues in Experiment 2, we no longer found a significant (cluster-corrected) gaze bias in the null-retrocue blocks (cluster p = 0.1978), though the gaze biases following informative pro- and anti-retrocues were each still pronounced (**Fig. S2d**; cluster ps < 0.0001). In line with the absence of a gaze bias following null-retrocues, we also no longer observed a significant difference in gaze bias following pro- and anti-retrocues (no clusters found), nor a delay in the gaze bias following anti-retrocues (**Fig. S2d-e**; Jackknife-$t_{(24)}$ = -0.295, p = 0.770).

Nevertheless, when correlating the magnitudes of the putative orientation-match gaze bias and the pro-vs-anti gaze bias across time (r = 0.69, p < 0.001) and across participants (**Fig. S2f;** $r_{(23)}$ = 0.398; p = 0.055), we observed trends hinting at a qualitatively similar effects as those reported for Experiment 1 (**Fig. 3b-c**). Moreover, as in Experiment 1, we found that gaze bias predicted subsequent memory performance in several conditions of the experiment (**Fig. S3**).

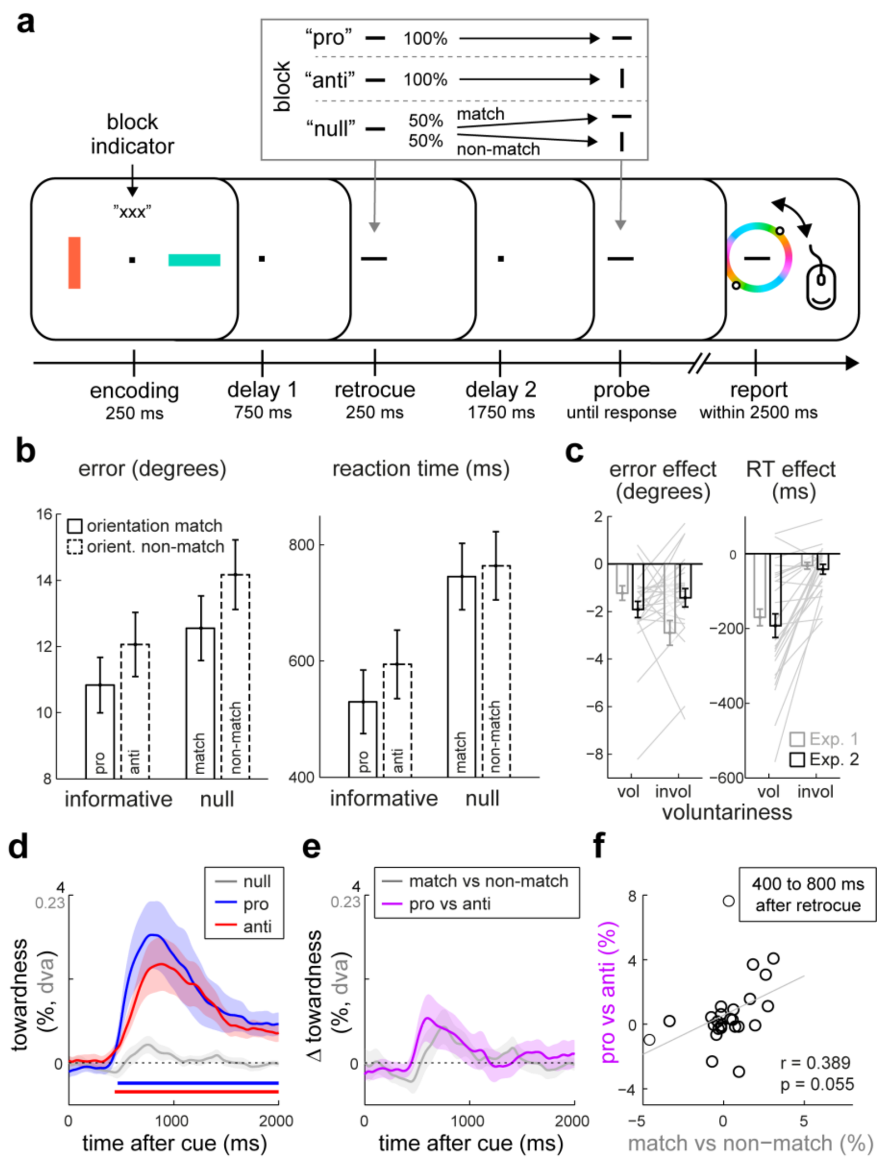

Figure S2. Orientation-retrocues yields less capture than colour-retrocues, in behaviour and in gaze. a) Schematic of working-memory task in Experiment 2. The task was identical to the task in Experiment 1 (**Fig. 1a**), except that memoranda were now cued and probed using orientations (horizontal or vertical), and participants had to reproduce memorised colour. **b)** Performance as a function of retrocue informativeness and orientation match. **c)** Effects of voluntary and involuntary attentional influences on behaviour. To aid visual comparison, light-grey bars repeat the corresponding data from Experiment 1. Grey lines show attentional effects in individual participants. Note the drop in the involuntary capture effect on error in Experiment 2, despite the similar voluntary effect on RT. **d)** Overlay of gaze-bias towardness time courses following null-, pro-, and anti-retrocues. **e)** Overlay of relevant gaze-bias differences. **f)** Correlation across participants between relevant gaze-bias differences. Error bars and shaded areas represent ± 1 SEM, calculated across participants (n = 25).

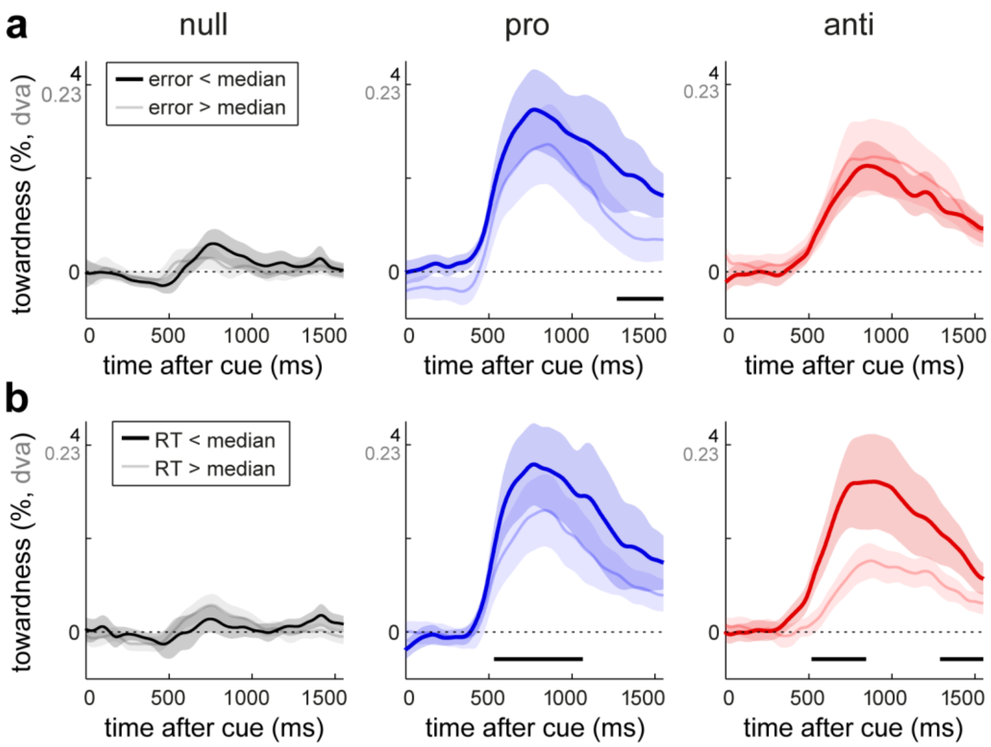

Figure S3. Gaze bias as a function of subsequent performance in Experiment 2. Identical to Figure 4, except showing gaze bias split by error (panel **a**) or reaction time (panel **b**) following null-, pro-, and anti-retrocues in Experiment 2.

Supplementary discussion: Experiment 1 vs. Experiment 2

Thus, we found a particularly clear capture effect when colour was used to retrocue memory items (in Experiment 1), but much weaker capture effects when using orientation-based retrocues instead (in Experiment 2) – despite similar voluntary effects between Experiments. We were able to use this "natural variation" between experiments to our advantage. Following orientation retrocues in Experiment 2, we no longer observed a significant pure-capture-related gaze bias to the orientation-matching memory item (in null retrocue blocks), and also no longer observed a delay in the gaze bias following anti-retrocues (despite the clear manifestation of gaze bias following anti-retrocues in Experiment 2). This corroborated our suggestion that the delayed gaze bias following colour anti-retrocues in Experiment 1 reflects the competition between voluntary and involuntary factors, rather than some peculiarity of using anti-retrocues per se (which should have yielded a delayed gaze bias in both experiments).

Nevertheless, clarifying the factors that contribute to the observed differences between the automatic influences of colour and orientation retrocues on memory and gaze remains an important avenue for future research. For example, unlike the orientation retro-cues that we used in experiment 2 – which are inherently spatial – colour is a non-spatial property. Accordingly, colour may be considered a more global (spatially independent) feature by the brain – possibly making it easier for the colour of an item at one location to influence a matching item at another location (including in memorised visual space). Tentative support for this speculative interpretation comes from a prior study from our lab where we also found that attention to colour is more likely to "spread" to other items in visual working memory than attention to shape (see Experiment 3 in Niklaus et al., 2017).

M. Niklaus, A. C. Nobre, F. van Ede, Feature-based attentional weighting and spreading in visual working memory. *Sci. Rep.* **7**, 1–10 (2017).

Supplementary Figure 4

In addition to pro-, anti, and null-retrocues, we had also included “neutral” retrocues in a minority of trials (25%). Like our null retrocues, neutral retrocues were also uninformative. However, unlike our null retrocues, our neutral retrocues did not match the content of memory in colour (experiment 1) or orientation (experiment 2), but instead involved a colour change to grey (experiment 1) or a combination of horizontal and vertical orientations (experiment 2), as depicted on the right in **Figure S4**. These trials were left out of our main analyses because neutral retrocues had no place in our two-by-two operationalisation of voluntary and involuntary factors and because our gaze measure was not defined relative to neutral cues that had no relation to left/right memory items. However, for transparency and completeness, we present the behavioural data from these trials alongside the data from our four main experimental conditions in **Figure S4** below.

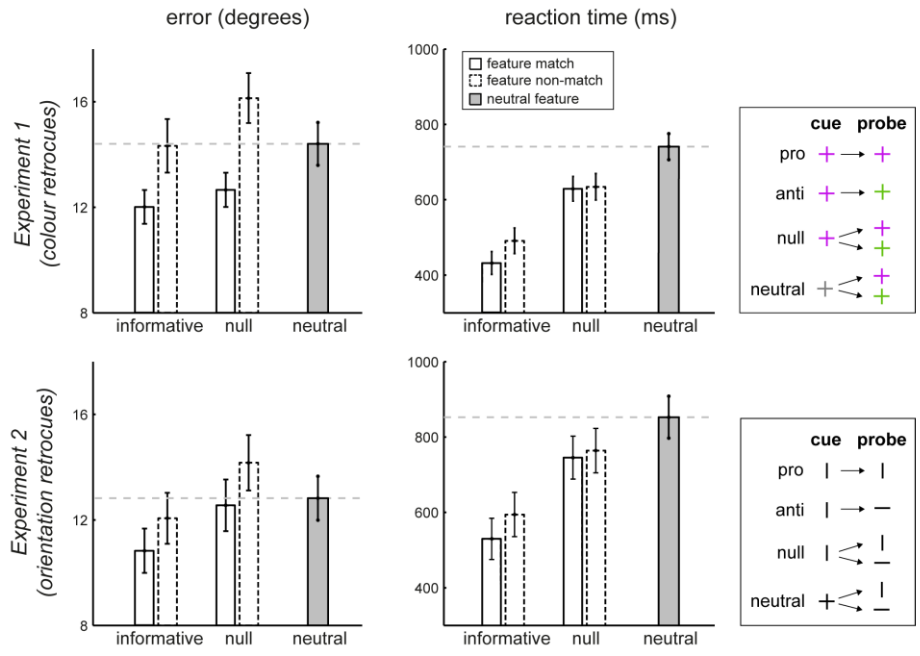

Figure S4. Behavioural data from the neutral-retrocue conditions alongside our four main conditions in experiments 1 (top) and 2 (bottom), for reproduction error (left) and reaction time (right).

Response errors in the neutral trials followed a sensible pattern. Relative to trials with neutral retrocues, this showed, for example, better performance in trials with matching retrocues and worse performance in trials with non-matching retrocues (especially in Experiment 1 where this capture effect on errors was more clearly evident). However, we also noted that reaction times were always slower in trials with neutral retrocues. We believe this may be related to at least two aspects. First, neutrals trials were relatively infrequent in a block and may have therefore been relatively confusing/surprising to participants. Second, in neutral trials, the cue was somewhat different in visual appearance than in all of the other trials, which may have been another potential source of confusion. Such confusion may have slowed RTs in these trials, and warrants some caution when comparing the data against this particular condition. In hindsight, this condition may thus have been too distinct to enable an informative comparison to the other conditions. For this reason, we refrained from formal statistical comparisons against this “neutral” condition.
